# Huge Concha Bullosa Involved With Allergic Fungal Sinusitis and Mimicking a Bony Tumor

**DOI:** 10.7759/cureus.15586

**Published:** 2021-06-11

**Authors:** Saud R Alromaih, Nouf S Aloraini, Saleh K Alqaryan

**Affiliations:** 1 Otolaryngology - Head and Neck Surgery, King Saud University, Riyadh, SAU; 2 Otolaryngology - Head and Neck Surgery, King Saud Medical City, Riyadh, SAU

**Keywords:** conchal pneumatization, chronic rhinosinusitis, nasal polyposis, mri

## Abstract

Concha bullosa (CB) is a pneumatized air cell within the nasal turbinates, often in the middle turbinate. CB is one of the most common anatomical variations found in the middle turbinate. It generally tends to be asymptomatic but can cause symptoms such as nasal obstruction, facial pain, and a decrease in the sense of smell. Moreover, CB can be involved in chronic rhinosinusitis, which can lead to its confusion with other lesions, as the radiographic picture might mimic other nasal pathologies. Here, we report a case of massive CB involved with allergic fungal sinusitis (AFS) and mimicking a bony tumor in order to highlight the diagnostic challenge of this clinical entity.

## Introduction

Concha bullosa (CB) is the most common anatomical variation of the middle turbinate. It is defined as pneumatization of the middle turbinate, and its incidence varies from 14% to 53%. CB can be unilateral or bilateral. CB is commonly asymptomatic and is usually an incidental finding on computed tomography (CT). However, CB may cause symptoms such as nasal obstruction, facial pain, and a decrease in the sense of smell [1,2]. Typically, CB contains mainly air; it is very rare to have a fungal mass within the CB, which has only occurred in a limited number of reported cases [3,4]. However, fungal involvement of the nasal and paranasal sinuses has increased in recent years, with Aspergillus species being the most common isolated fungus [5]. In this article, we present a case of massive CB that was involved with allergic fungal sinusitis (AFS) and which mimicked a sinonasal bony lesion on radiographic pictures. 

## Case presentation

A healthy 20-year-old female came to our clinic with a complaint of progressive, continuous, and long-standing history of bilateral nasal obstruction that was worse on the left side. She was having frequent thick mucoid nasal secretions, left-sided facial pain, and intermittent hyposmia, also it was associated with moderate to severe allergic rhinitis symptoms; she had no visual disturbances. The patient had already tried intranasal corticosteroids with no satisfactory improvement. 

**Figure 1:**
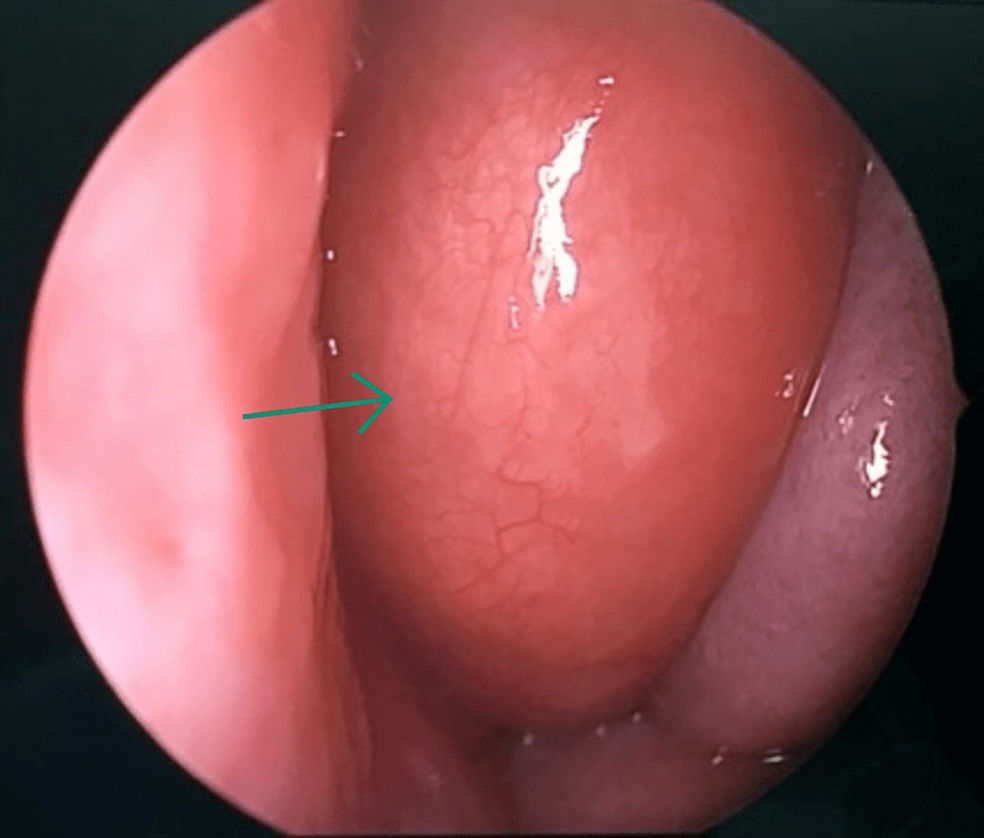
Endoscopic view of the mass, which is covered with smooth, healthy mucosa.

A nasal endoscopic exam revealed a deviated nasal septum to the right side, a mildly hypertrophied right inferior turbinate, and a large mass occupying the left nasal cavity. This mass had displaced the nasal septum to the contralateral side and compressed the left inferior turbinate. Using the freer and fraiser suction, the mass was found to be hard but with normal overlying mucosa (Figure 1).

It originated from the skull base on the endoscope, with small nasal polyps noted beside it, but a further passage to the nasal cavity was not possible. The rest of the clinical exam, including her cranial nerves and orbital exam, was unremarkable. 

The patient came to our clinic for a second opinion, as she was concerned about the results of her medical imaging, which included CT scans and magnetic resonance imaging (MRI) of the paranasal sinuses. These reports commented on a bony nasal lesion measuring 1.7 × 3.7 × 3.0 cm and arising from the left skull base. The lesion was obstructing all ipsilateral paranasal sinuses with complete opacification and displacing the nasal septum to the contralateral side. Fortunately, there was no intracranial or orbital extension. No comments were made regarding heterogeneous opacification. These findings were thought to be an aneurysmal bone cyst, mucocele, mucopyocele, or other sinonasal tumors. 

**Figure 2:**
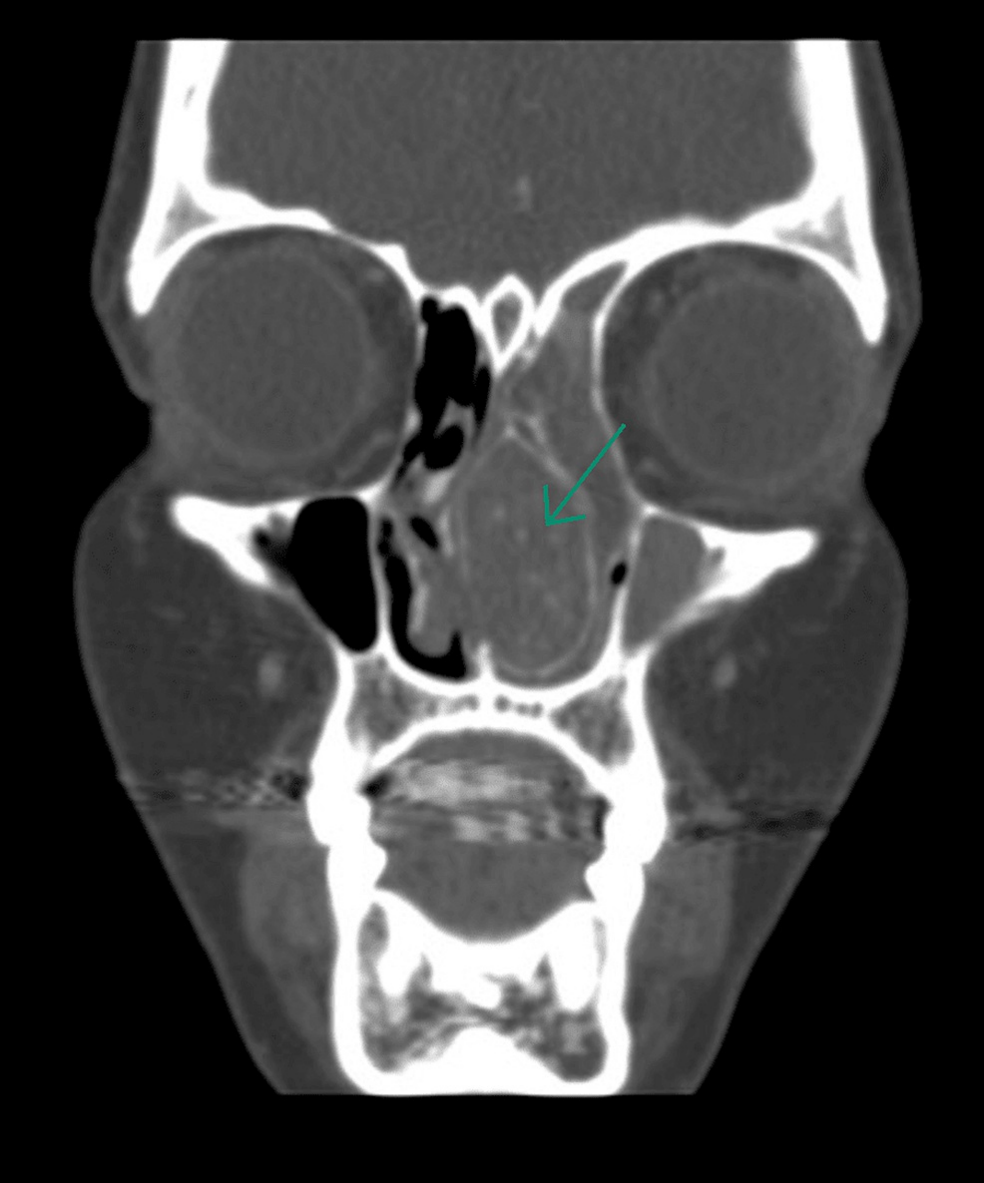
Coronal section of bone-window CT showing an expansile mass of 1.7 × 3.7 × 3.0 cm with central calcification and egg-shell border. The mass is obstructing the left nasal airway and causing compression over the septum.

**Figure 3:**
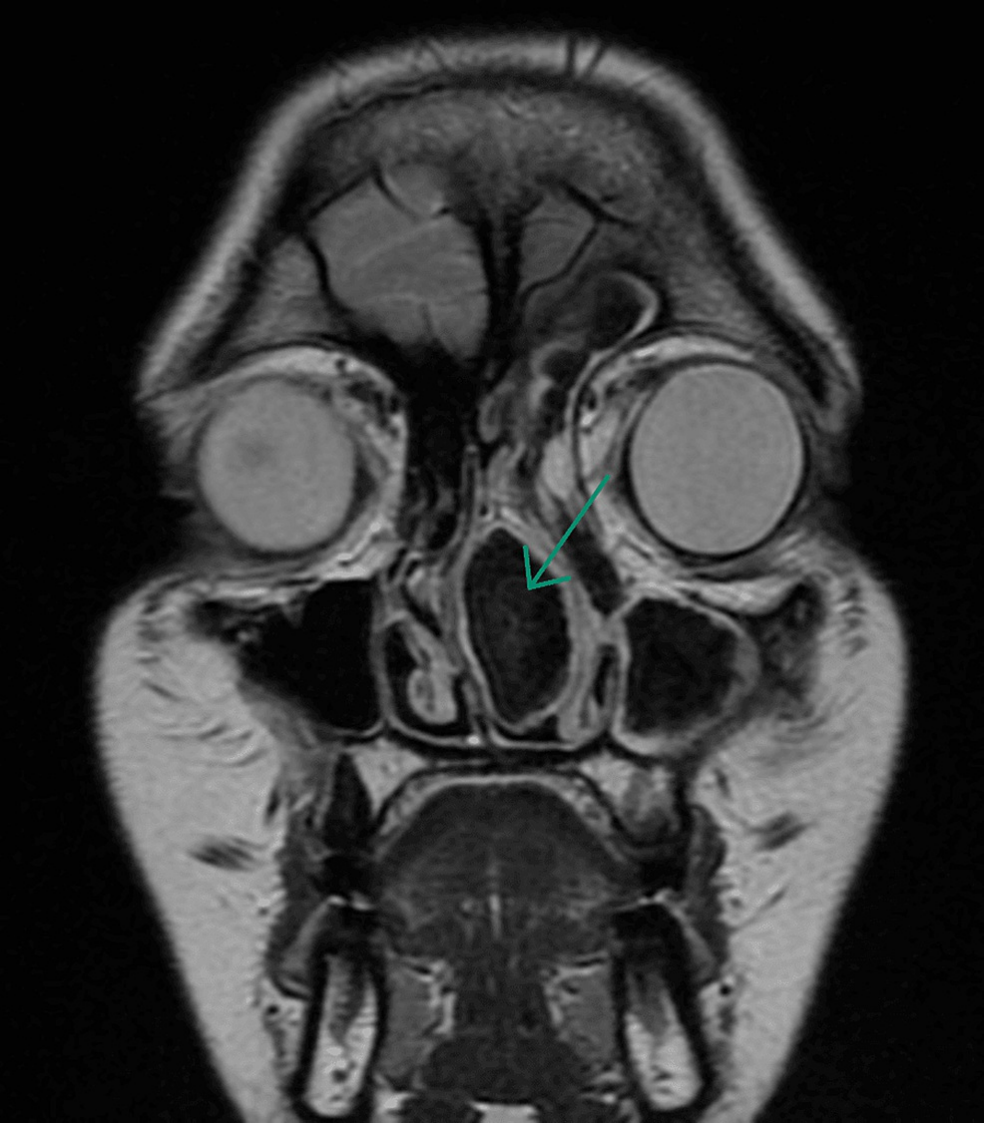
T2-weighted MRI showing the low signal intensity of the mass.

However, upon careful study of the imaging, AFS was considered due to the heterogeneous nature of the content, the bony expansion of the lesion in the CT scans, the low signal intensity on T2-weighted images, and the heterogeneous high signals on T1-weighted images (Figures 2-3).

She was then assured and counseled for surgery. The patient had a routine and unremarkable preoperative assessment and then underwent functional endoscopic sinus surgery, polypectomy, and septoturbinoplasty (resecting whole middle turbinate) under general anesthesia. 

**Figure 4:**
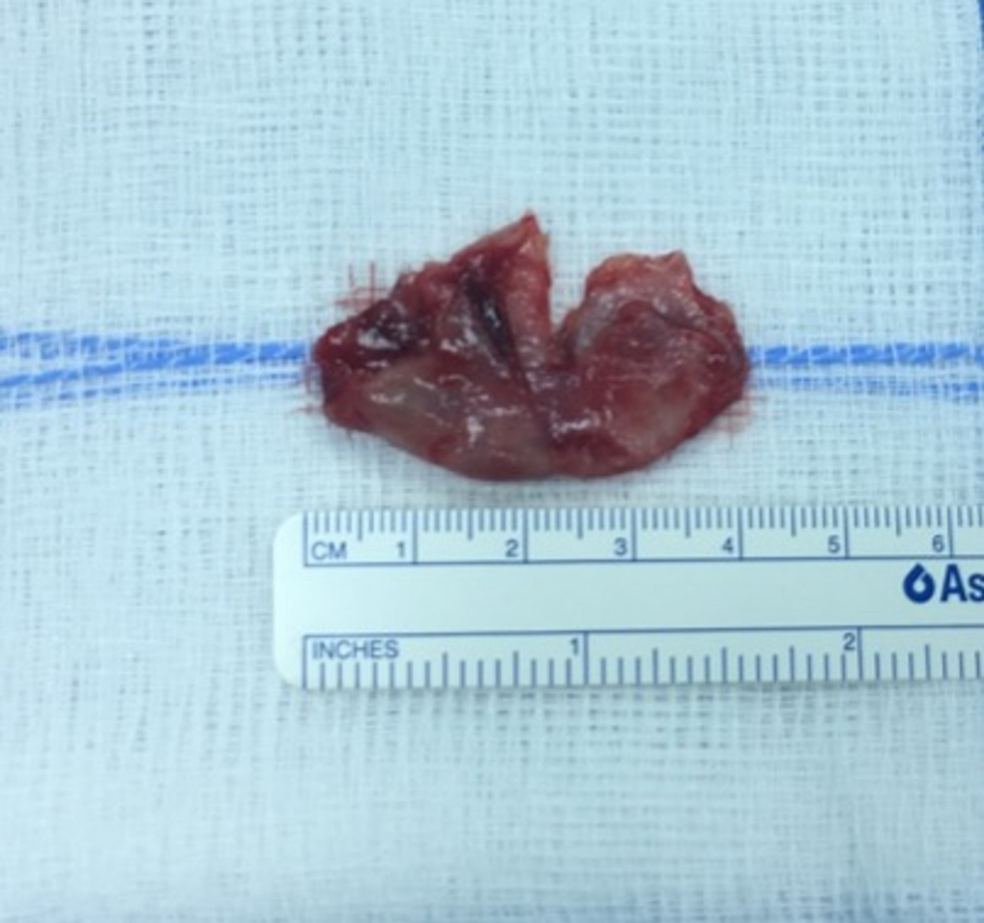
Mass post resection.

Intraoperatively, the mass was found to be a huge CB of the left middle turbinate that was filled with debris. Biopsies were taken from its mucosa and debris for histopathological staining and regular culture. It was resected, and all ipsilateral sinuses were opened and drained. The skull base and orbital wall were intact, and the surgery was uneventful (Figure 4). 

The patient was given a tapered dose of oral steroids over 15 days and had an uneventful post-operative course. 

The histopathologic examination revealed amorphous debris with focal calcification and inflammatory cells with no evidence of malignancy. The material sent for fungal stain was reported positive. The intraoperative nasal swab result showed no pathogenic bacteria isolated after 48 hours of incubation at 37°C. In the subsequent postoperative visits, the patient remained asymptomatic, and the endoscopic exam showed a patent nasal airway, healthy mucosa, and no signs of recurrence.

## Discussion

Bolger et al. classified CB according to its anatomical location: lamellar when it involves the vertical lamella and bulbous when it involves the inferior bulbous. If the pneumatization involves the whole concha, it is described as extensive [1]. As noted previously, the common symptoms of CB are nasal obstruction, headache, and a decrease in the sense of smell, which were consistent with our patient’s presentation. The relationship between sinusitis and CB is still controversial, as there are conflicting data on whether CB could be a cause of sinusitis [6]. Marianowski et al. described four surgical techniques for managing CB - lateral marsupialization, medial marsupialization, crushing, and transverse excision [7].

Fungal rhinosinusitis (FRS) is generally classified into invasive and non-invasive, according to the mucosal layer invasion. AFS is considered to be a non-invasive type of FRS. AFS is defined largely by the presence of basal polyposis with allergic mucin, that is, thick, tenacious, and eosinophilic secretions with characteristic histologic findings. The endoscopic finding associated with AFS is nasal polyposis, which generally affects multiple sinuses with a unilateral predominance. Furthermore, radiological features found in AFS on CT have been described as intrasinus serpiginous areas of increased attenuation causing heterogeneous images with bony expansion and thinning. Additionally, MRIs have demonstrated very hypointense regions surrounded by mucosal inflammation [8,9]. 

Bent and Kuhn established the standard criteria for the diagnosis of AFS. These criteria are based on clinical, radiological, immunological, and histological characteristics of the disease, including 1) type I hypersensitivity confirmed by history, skin tests, or serology; 2) nasal polyposis; 3) characteristic CT features; 4) eosinophilic mucus without fungal invasion; 5) positive fungal stain of sinus contents removed intraoperatively [8]. The current treatment of choice in AFS patients is surgical, consisting of functional endoscopic sinus surgery with debridement of impacted mucin and fungal debris along with aeration of the diseased sinuses. Moreover, medical therapies play an important role in the post-operative course, as described by Gan et al. Medical therapies used in AFS consist of continued topical corticosteroids as well as a short course of oral steroids, which decrease the inflammatory response, eosinophilia, and IgE levels. The use of oral antifungals and immunotherapy is reserved for select limited cases [10].

In this case, the main cause of confusion was the radiological readings, which were misinterpreted as other nasal lesions. Thus, the possibility of AFS involvement was not considered due to the lack of experience in this area. We would like to highlight the importance of clinicians carefully reading the images provided and combining them with clinical knowledge of differential diagnoses to avoid such confusion.

## Conclusions

This case report highlights the importance of including the diagnosis of AFS in CB. Radiological modalities can show bony expansions that may be confused with neoplasm of the nasal cavity. Endoscopic surgical treatment can confirm the diagnosis of AFS and is usually curative.
